# Causal effects of maternal BMI on pregnancy outcomes: a Mendelian randomisation study investigating the mediating role of blood counts

**DOI:** 10.3389/fgene.2026.1697926

**Published:** 2026-04-24

**Authors:** Christopher Flatley, Geng Wang, Alesha Hatton, Kym-Mai Nguyen, Liang-Dar Hwang, Nicole M. Warrington

**Affiliations:** 1 Institute for Molecular Bioscience, University of Queensland, Brisbane, QLD, Australia; 2 Frazer Institute, University of Queensland, Brisbane, QLD, Australia

**Keywords:** birth weight, blood counts, body mass index, genetics, gestational age, gynaecology, mediation, Mendelian randomisation

## Abstract

**Background:**

Pregnancy requires a delicate balance between the maternal immune system and inflammatory responses. Elevated maternal body mass index (BMI) significantly compromises the immune system and increases systemic inflammation. High maternal BMI is associated with adverse pregnancy outcomes, including an increased risk of both pre-eclampsia and preterm birth, which may be mediated through immune-related blood cell changes.

**Methods:**

This study used Mendelian randomisation (MR) to investigate the causal relationship between maternal BMI and pregnancy outcomes, including birth weight, placental weight, gestational duration, and pre-eclampsia. We applied two-step MR to assess whether immune-related blood counts, such as neutrophils, lymphocytes, and platelets, mediate these relationships. Single-nucleotide polymorphism (SNP) effect estimates for maternal BMI and pregnancy outcomes were sourced from publicly available genome-wide association studies (GWASs), with pregnancy outcomes partitioned into maternal genetic effects to proxy genetic effects on the intrauterine environment.

**Results:**

We found that elevated maternal BMI causally increased placental weight (β_IVW_ = 0.164 standard deviation (SD) increase in placental weight per SD increase in maternal BMI, *p* = 2.92 × 10^−7^) and the risk of pre-eclampsia (OR_iVW_ 1.75, *p* = 6.3 × 10^−30^). The effect of maternal BMI on placental weight was larger than its effect on birth weight. Mediation analysis found no evidence of the involvement of immune-related blood counts in these relationships.

**Conclusion:**

Maternal BMI has a significant impact on pregnancy outcomes, particularly by increasing placental weight and the risk of pre-eclampsia. These findings highlight BMI-driven placental adaptations as key contributors to pregnancy complications.

## Introduction

While maternal body mass index (BMI) typically increases during pregnancy, there is now a global trend of higher pre-pregnancy BMI among women of childbearing age (Ovesen et al., 2011; Creanga et al., 2022). Elevated maternal BMI is associated with adverse pregnancy outcomes. Pre-pregnancy obesity (defined as BMI greater than 25 kg/m^2^) significantly increases the risk of pre-eclampsia (odds ratio (OR) 3.01, 95% CI: 2.86, 3.17) (Ovesen et al., 2011), fetal macrosomia (OR 2.28, 95% CI: 2.15, 2.41) (Creanga et al., 2022), and preterm birth (1.17, 95% CI: 1.13, 1.21) (Creanga et al., 2022).

Pregnancy relies on a delicate equilibrium between the maternal immune system and inflammatory responses, pivotal for its successful progression from ovulation to labour onset. This process begins with local inflammatory reactions triggered by blastocyst-endometrial contact, followed by decidualisation, which starts during the menstrual cycle. Decidualisation allows endometrial stromal cells to evaluate blastocyst quality through changes in their secretory profile (Salker et al., 2010; Fernández et al., 2022). This is followed by the release of pro-implantation factors and immunological regulation, particularly involving lymphocytes and neutrophils, which are essential for a successful pregnancy (Jafarpour et al., 2020; Bert et al., 2021). Though physiological increases in white blood counts are common during pregnancy, excessive alterations may precipitate pregnancy complications (Akgun et al., 2017; Morisaki et al., 2021; Zhang et al., 2023).

Elevated BMI is associated with systemic chronic inflammation (Creanga et al., 2022; Garske et al., 2023), leading to shifts in immune-related blood cell populations, including monocytes, neutrophils, and lymphocytes (Artemniak-Wojtowicz et al., 2020). Macrophages, derived from monocytes, can constitute up to 40% of obese adipose tissue compared to less than 10% in individuals with a normal BMI (Purdy and Shatzel, 2021). Neutrophils, the most abundant white blood cells (WBCs), act the first responders in the inflammatory response. In obese patients, they infiltrate adipose tissue (unlike in lean individuals, where they are absent from adipose tissue during haemostasis), and therefore, elevated neutrophil counts are exhibited, which release significantly more pro-inflammatory mediators (Gomez-Casado et al., 2024).

Leukocytosis, a higher-than-normal WBC count, is a normal physiological adaptation during pregnancy. However, increased WBC counts in pregnancy have been associated with the development of pre-eclampsia (OR 1.14, 95% CI: 1.47, 1.64), preterm birth (OR 1.12, 95% CI: 1.06, 1.18), and low birth weight (OR 1.12, 95% CI: 1.08, 1.16) (Zhang et al., 2023). In individuals with pre-pregnancy obesity, chronic low-grade inflammation may further amplify leukocytosis, potentially exacerbating the risk of these adverse pregnancy outcomes (Purdy and Shatzel, 2021; Zhang et al., 2023).

The pathophysiology of adverse pregnancy outcomes is intricate and multifaceted. Gaining insight into their aetiology and devising strategies to mitigate these outcomes necessitates understanding the causal pathways leading to these outcomes and identifying potentially modifiable risk factors. However, observational association studies are hindered by confounding and reverse causation, which weakens their ability to provide strong evidence of causality. Mendelian randomisation (MR) is a method used to infer causal relationships between a modifiable exposure and a relevant disease or trait, using genetic variants (typically single-nucleotide polymorphisms [SNPs]) as instrumental variables (Smith and Ebrahim, 2003; Sanderson et al., 2022). At the core of MR lie three assumptions that must be met to have valid instrumental variables: the genetic variants must be associated with the modifiable exposure (relevance), there are no confounding variables (measured or unmeasured) between the genetic variants and the outcome (independence), and the genetic variants exclusively impact the outcome through the modifiable exposure (exclusion restriction) (Davies et al., 2018; Burgess et al., 2019). The most commonly used framework, known as two-sample MR, uses SNP-exposure and SNP-outcome estimates from two separate samples (often the largest genome-wide association studies of the traits) to estimate the causal effect of the exposure on the outcome. Two-step MR allows the investigation of the mediating effects of an intermediate variable within a causal modelling framework (Relton and Davey Smith, 2012; Burgess and Thompson, 2015).

Pre-eclampsia has been linked to both cardiometabolic dysregulation and altered systemic inflammatory responses in pregnancy (Burton et al., 2019; Purdy and Shatzel, 2021; Zhang et al., 2023; Ardissino et al., 2024). A recent MR study demonstrated that elevated BMI is causally associated with an increased risk of pre-eclampsia (OR 1.68, 95% CI: 1.46, 1.94, *p* = 8.74 × 10^−13^) (Ardissino et al., 2024). Tyrrell et al. (2016) demonstrated a causal association between maternal BMI and offspring birth weight, showing that a one standard deviation increase in maternal BMI was associated with a 55-g increase in birth weight (95% CI: 17, 93 g) (Tyrrell et al., 2016). An MR study investigating BMI and blood traits found a negative causal association between BMI and both WBC and platelet count (Thom et al., 2022). Additionally, lymphocyte count is causally associated with pre-eclampsia (OR 1.10, 95% CI: 1.01, 1.21) (Zeng et al., 2024). Finally, a study examining various leukocyte subsets identified causal relationships between specific immune cell populations and both birth weight and risk of preterm birth (Chen et al., 2024). These findings indicate complex relationships between maternal BMI, blood counts, and various pregnancy outcomes.

The causal relationships identified in previous MR studies support the hypothesis that blood counts may play a mediating role in the relationship between maternal BMI and pregnancy outcomes. Therefore, this study aims to use MR to investigate the causal relationship between maternal BMI and pregnancy outcomes, including birth weight, placental weight, gestational duration and pre-eclampsia and hypertensive disorders. If a causal relationship exists, we will use a two-step MR to investigate whether blood counts—including basophil, eosinophil, lymphocyte, monocyte, neutrophil, and platelet counts—mediate this relationship.

## Methods

### Instrumental variables

#### Body mass index

We extracted summary data from the largest European genome-wide association study (GWAS) on BMI to date, which included approximately 700,000 individuals (Yengo et al., 2018). This meta-analysis combined GWAS results for BMI from the United Kingdom Biobank (UKBB) and the GIANT consortium: BMI was analysed on an inverse-normal-transformed (standardised) scale in the UKBB component, and effect estimates are, therefore, interpreted per standard-deviation increase in BMI (Yengo et al., 2018). To identify SNPs to use as instrumental variables, clumping was performed using the Two Sample MR package (Version 0.6.4) in R (Version 4.2.1) on the GWAS summary statistics, using *p* < 5 × 10^−8^, *r* ^2^ = 0.001 and a 10,000 kb window. This identified 521 independently associated genetic variants for BMI; however, due to some missing data in the blood count and pregnancy outcome GWASs, the number of genetic variants used as instruments for BMI in each analysis was slightly lower. The genetic variants, along with the mean F-statistic for each analysis, are provided in Supplementary Table S1.

### Mediator

#### Blood counts

Summary statistics for SNPs associated with blood count phenotypes (basophil, eosinophil, monocyte, lymphocyte, neutrophil, and platelet counts) were obtained from a large GWAS utilising the UKBB (Vuckovic et al., 2020). This study included 408,112 participants of European ancestry. Platelet counts were directly measured in the UKBB as the number of platelets per unit volume of blood using impedance. The remaining blood counts were derived, first as a percentage of WBCs and then converted to absolute counts using the formula (Vuckovic et al., 2020):

$$\text{Blood Count}=\frac{\text{Blood}\;\text{Cell}\%\times \text{WBC}\#}{100}$$

where WBC# represents the total WBC count and Blood Cell% represents the percentage of WBCs for that specific blood cell type.

Prior to GWASs, log_10_-transformed blood counts were adjusted for age, age squared, sex, principal components, and cohort-specific covariates, and residuals were inverse-normalised (Vuckovic et al., 2020).

To perform a two-step MR, two analyses are required using the blood counts. First, SNPs associated with these blood counts were used as instrumental variables to assess relationships between blood counts and pregnancy outcomes. Second, SNP–blood cell count estimates were used to investigate the causal relationship between BMI and blood counts. We employed the same clumping and threshold methods described for the BMI summary statistics to identify independent SNPs to use as instrumental variables for each blood cell count. The number of variants used as instruments for each blood cell count, along with the mean F-statistic, is provided in Supplementary Table S1.

### Outcome variables

Correlated maternal and fetal genomes both influence pregnancy-related measures, and therefore, conditional analyses are required to partition the genetic effect into maternal and fetal-specific components. The maternal-specific component is, therefore, the effect of the maternal genotype on the pregnancy-related measure that is independent of fetal genotype. A method, the weighted linear model (WLM), has been developed that takes the unadjusted genetic effect estimates from GWASs and transforms them into adjusted fetal- and maternal-specific genetic effects (Warrington et al., 2019). To avoid violating the assumptions underlying MR, the maternal-specific genetic effect was used in the current analyses to proxy maternal exposures during pregnancy (BMI and blood counts) (Evans et al., 2019).

#### Birth weight

Birth weight GWAS summary statistics were obtained for offspring birth weight (n = 270,002) and (own) birth weight (n = 423,683) from Juliusdottir et al. (2021). First, the DECODE birth weight measures were adjusted for offspring sex, year of birth, gestational age at birth, and maternal age, and then a rank-based inverse normal transformation was applied. A GWAS was then performed using BOLT-LMM (v2.1) (Loh et al., 2015). Subsequently, a meta-analysis was performed on both the offspring’s birth weight and their own birth weight, combining the DECODE summary statistics with previously published results from the EGG consortium and UKBB participants of European ancestry (Juliusdottir et al., 2021). To estimate maternal-specific genetic effects on offspring birth weight, we applied a weighted linear model (Warrington et al., 2019) to the summary statistics from this meta-analysis. To achieve this, we merged the offspring birth weight GWAS summary statistics with the fetal (own) birth weight summary statistics and derived the adjusted maternal effect (Warrington et al., 2019):

$$\hat{\beta }{}_{\text{Maternal}\;\text{Adj}}=\left(\frac{4}{3}\times \hat{\beta }{}_{\text{Maternal}}\right)-\left(\frac{2}{3}\times \hat{\beta }{}_{\text{Fetal}}\right)$$

where $\hat{\beta }{}_{\text{Maternal}}$ is the SNP effect size from the (unadjusted) GWASs of the maternal genome on offspring birth weight and $\hat{\beta }{}_{\text{Fetal}}$ is the SNP effect size from the (unadjusted) GWASs of the fetal genome on their own birth weight. For more information regarding the WLM, see *Weighted Linear Model to Partition the SNP Effect Estimates on Birth Weight* in the Supplementary Methods.

#### Placental weight

Placental weight summary statistics were sourced from Beaumont et al. (2023), who conducted a GWAS on placental weight using fetal (n = 65,405), maternal (n = 61,228), and paternal (n = 52,392) genotypes of European ancestry. The analysis accounted for fetal sex and gestational age, and outcomes were standardised to Z-scores to adjust for variability in collection methods (e.g., trimmed vs. untrimmed placentas). The study included placentas from births between 37 and 43 weeks of gestation, with placental weights ranging from 200 to 1,500 g. Beaumont et al. (2023) partitioned their results into maternal-specific effects using the WLM approach described above, which we utilised for this current study.

#### Gestational duration

GWASs of gestational duration were sourced from the fetal genome GWAS by Liu et al. (2019) (n = 84,689) and the maternal genome GWAS by Solé-Navais et al. (2023) (n = 195,555). Both GWASs were performed on subjects of European ancestry. The partitioned maternal-specific effect from these GWASs was obtained using the Direct and INdirect effects analysis of Genetic lOci (DINGO) method described by Hwang et al. (2023). This approach partitions the genetic effect into maternal and offspring-specific genetic components in a similar fashion to the WLM approach. Partitioned summary statistics are reported as standardised Z-scores (Hwang et al., 2023).

#### Pre-eclampsia and hypertensive disorders of pregnancy

A pre-eclampsia and hypertensive disorders of pregnancy GWAS of European ancestry was conducted by Tyrmi et al. (2023) (cases = 15,200, controls = 115,007). Cases for the pre-eclampsia and hypertensive disorders of pregnancy phenotype were based on International Classification of Diseases codes from ICD-10 (O10, O11, O13, O14, 015, and O16), ICD-9 (642) and ICD-8 (63701, 63703, 63704, 63709, 63710, 63799, and 66120), and parous women without these codes were classified as controls (Tyrmi et al., 2023). We used the SNP-effect sizes from this GWAS for our MR analyses.

### Two-sample Mendelian randomisation

Two-sample MR estimates a causal effect of an exposure on an outcome by calculating a Wald ratio for each instrumental variable (here, we use SNPs), which is the ratio of the SNP-outcome association to the SNP-exposure association (Lawlor, 2016). These Wald ratios are then meta-analysed using a multiplicative random-effects inverse-variance-weighted (IVW) approach to provide an overall causal estimate (Hemani et al., 2017; Hemani et al., 2018a). We performed IVW analyses to assess whether there is a causal relationship between maternal BMI, blood counts, and pregnancy outcomes (birth weight, placental weight, gestational duration, and pre-eclampsia).

Horizontal pleiotropy, where the instrumental variable is related to the outcome through a path other than the exposure, is a concern in MR studies as it violates the exclusion restriction assumption (Lawlor, 2016). Therefore, in addition to the IVW method, we also performed sensitivity analyses using multiple pleiotropy-robust MR models (MR-Egger, weighted median, simple mode, and weighted mode) that have varying assumptions regarding horizontal pleiotropy (Burgess et al., 2019). Cochran’s Q test was used to assess the heterogeneity of causal estimates across the different instrument variables.

All two-sample MR models were performed in R (Version 4.2.1) using the Two Sample MR Package (Version 0.6.4) (Hemani et al., 2018b). We used a threshold of *p* < 0.05 to determine putative causal relationships for inclusion in the two-step MR mediation analysis. A Bonferroni correction was applied to determine causal significance in the two-sample MR analysis of pregnancy outcomes, accounting for the seven exposures (blood counts and BMI), with the significance threshold set at *p* < 0.007.

### Two-step Mendelian randomisation

**FIGURE 1:**
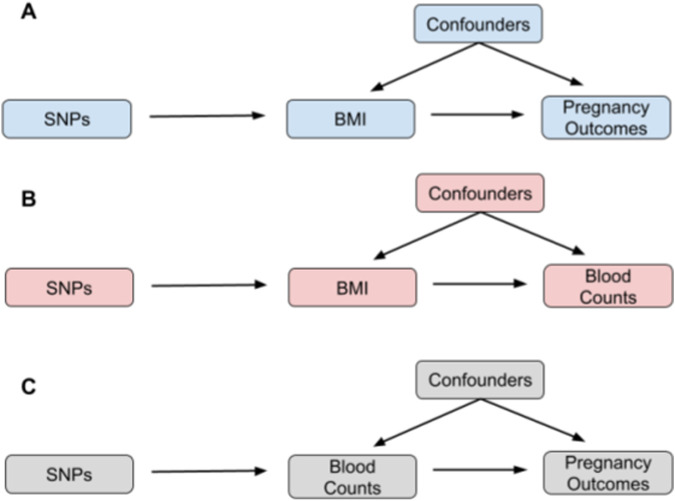
Putative causal pathways investigated using two-step Mendelian randomisation. **(A)** Pathway for the causal effect of maternal BMI on pregnancy outcomes; **(B)** pathway for the causal effect of maternal BMI on blood counts; **(C)** pathway for the causal effect of blood counts on pregnancy outcomes. SNPs: single nucleotide polymorphisms; BMI: maternal body mass index.

Two-step MR extends the classical two-sample MR framework by incorporating an additional step to assess mediation (Relton and Davey Smith, 2012). After confirming that a causal relationship exists between the exposure (maternal BMI) and outcome (pregnancy outcomes; Figure 1A), two-step MR tests if a causal relationship exists between the exposure of interest (e.g., BMI) and a mediator (e.g., blood counts; Figure 1B) and between the mediator (blood counts) and the outcome of interest (e.g., pregnancy outcomes; Figure 1C).

In mediation analysis, three statistics are of interest: the total effect, the direct effect, and the indirect effect. The total effect represents the univariate causal effect of BMI on pregnancy outcomes as derived from the two-sample MR (Figure 1A). The indirect effect captures the influence of BMI on pregnancy outcomes, which occurs solely through the mediating blood count trait. The direct effect is the estimated effect of BMI on pregnancy outcomes after accounting for any indirect effects. To estimate the indirect mediation effect using two-step MR, we apply the product of coefficient method, which multiplies the causal effect of the exposure on the mediator (Figure 1B) by the causal effect of the mediator on the outcome (Figure 1C). To isolate the direct effect of BMI on pregnancy outcomes, we can subtract the indirect effect from the total effect. The standard error for the indirect effect was derived using the delta method (Lynch and Walsh, 1998):

$${\hat{SE}}_{ab}^{\text{​}}=\sqrt{\left({\hat{b}}^{2}\times {\hat{SE}}_{a}^{2}\right)+\left({\hat{a}}^{2}\times {\hat{SE}}_{b}^{2}\right)+\left({\hat{SE}}_{a}^{2}\times {\hat{SE}}_{b}^{2}\right)}$$

where the indirect effects are as follows:

-
${\hat{a}}^{2}$ is the estimated causal effect of the exposure (BMI) on the mediator (blood count).
-
${\hat{b}}^{2}$ is the estimated causal effect of the mediator (blood count) on the outcome (pregnancy outcome).
-
${\hat{SE}}_{a}^{\text{​}}$ is the standard error of the estimated causal effect of the exposure on the mediator.
-
${\hat{SE}}_{b}^{\text{​}}$ is the standard error of the estimated causal effect of the mediator on the outcome.

The standard error for the direct effect is taken from the difference of two estimates method (MacKinnon, 2012):

$${\hat{SE}}_{direct}=\sqrt{{\hat{SE}}_{total}^{2}+{\hat{SE}}_{indirect}^{2}}$$

where ${\hat{SE}}_{total}^{2}$ is the standard error of the total effect of exposure on the outcome and ${\hat{SE}}_{indirect}^{2}$ is the standard error of the indirect effect (see above).

The *p*-value was calculated as a two-tailed probability from a Z-distribution. We calculated the total, direct, and indirect effect if a causal relationship (*p* < 0.05) was detected between the exposure, mediator, and outcome (i.e., if a causal effect was detected between BMI and birth weight, BMI and eosinophil counts, and eosinophil counts and birth weight, then the total, direct, and indirect effects were calculated). Although we acknowledge that *p* < 0.05 is a liberal threshold and may increase the number of false-positive associations in subsequent mediation analyses, we considered it necessary to restrict the number of mediation analyses performed (i.e., rather than performing mediation analyses on all combinations of mediators and outcomes).

## Results

### Effect of BMI on pregnancy outcomes

**FIGURE 2:**
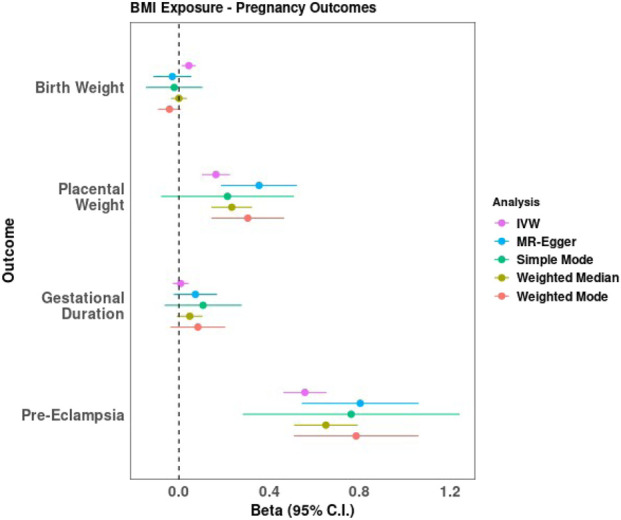
Results from two-sample Mendelian randomisation analyses of BMI on pregnancy outcomes.

Higher maternal BMI was found to causally increase offspring birth weight (*β*_IVW_ = 0.04SD increase in offspring birth weight per SD increase in maternal BMI, 95% CI: 0.01, 0.07, *p* = 0.005), but significant heterogeneity was detected (P_het_ = 2.79 × 10^−68^). The 95% confidence intervals around the causal effect estimates using the pleiotropy-robust methods (weighted mode, weighted median, and MR-Egger) overlapped with those from the IVW but were wider and crossed the null (Figure 2; Supplementary Table S2). Both the heterogeneity and lack of causal effect in the pleiotropy-robust methods suggest potential bias from horizontal pleiotropy.

IVW analysis indicated a causal association between elevated maternal BMI and increased placental weight (*β*_IVW_ = 0.16 SD increase in placental weight per SD increase in maternal BMI, 95% CI: 0.10, 0.23, *p* = 2.92 × 10^−7^). Similar to birth weight, significant heterogeneity was detected (P_he t_ = 7.47 × 10^−7^), and the MR Egger intercept indicated directional pleiotropy (*p* = 0.02; Supplementary Table S2). However, the 95% confidence intervals from the pleiotropy-robust methods overlapped with those from the IVW analyses, and the causal effect of elevated maternal BMI on increased placental weight remained after accounting for horizontal pleiotropy (Figure 2; Supplementary Table S2).

No significant causal relationship was observed between maternal BMI and gestational duration (*β*_IVW_ = 0.01 SD increase in gestational duration per SD increase in maternal BMI, 95% CI: −0.03, 0.04, *p* = 0.67). Again, significant heterogeneity was detected (P_het_ = 0.03), and the pleiotropy-robust methods also show no evidence of a causal effect (Figure 2; Supplementary Table S2).

Finally, a causal association was observed between maternal BMI and the risk of pre-eclampsia and hypertensive disorders of pregnancy (OR 1.75, 95% CI: 1.59, 1.92, *p* = 6.33 × 10^−30^), indicating that a higher BMI increases the risk of developing pre-eclampsia and hypertensive disorders during pregnancy. While there was significant heterogeneity (P_het_ = 1.18 × 10^−6^) and the MR-Egger intercept suggested directional pleiotropy (*p* = 0.05), the pleiotropy-robust methods support the finding of causality after accounting for horizontal pleiotropy (Figure 2; Supplementary Table S2).

### Effect of BMI on blood counts

The IVW results provided evidence that higher maternal BMI causes a lower basophil count (*β*_IVW_ = −0.02 SD decrease in basophil count per SD increase in maternal BMI, 95% CI: −0.05, −0.002, *p* = 0.03), eosinophil count (*β*_IVW_ = −0.04 SD decrease in eosinophil count per SD increase in maternal BMI, 95% CI: −0.08, −0.004, *p* = 0.03), monocyte count (*β*_IVW_ = −0.07 SD decrease in monocyte count per SD increase in maternal BMI, 95% CI: −0.10, −0.03, *p* = 9.80 × 10^−5^) and platelet counts (*β*_IVW_ = −0.07 SD decrease in platelet count per SD increase in maternal BMI, 95% CI: −0.12, −0.03, *p* = 0.001). We detected significant heterogeneity for all of these causal effects, and the estimated causal effects attenuated towards the null when using approaches more robust to horizontal pleiotropy (Supplementary Figure S1; Supplementary Table S3). BMI showed no causal relationship with lymphocyte count (*p* = 0.23) or neutrophil counts (*p* = 0.30, Supplementary Figure S1; Supplementary Table S3).

### Effect of blood counts on birth weight

Of the blood counts, both elevated eosinophil count (*β*_IVW_ = −0.03 SD decrease in offspring birth weight per SD increase in eosinophil count, 95% CI: −0.05, −0.01, *p* = 0.01) and elevated lymphocyte count (*β*_IVW_ = −0.04 SD decrease in offspring birth weight per SD increase in lymphocyte count, 95% CI: −0.06, −0.02, *p* = 0.001) were found to have a causal relationship with lower birth weight. Significant heterogeneity was again detected for both causal relationships (P_het_ = 5.62 × 10^−30^, P_het_ = 4.47 × 10^−35^ for eosinophil and lymphocyte counts, respectively). The pleiotropic-robust methods showed consistent results with the IVW causal estimates, although the standard errors were slightly larger (Supplementary Figure S1; Supplementary Table S4). No other blood counts were found to have causal relationships with birth weight (Supplementary Figure S1; Supplementary Table S4).

### Effect of blood counts on placental weight

No causal relationships were observed between any of the blood counts and placental weight (Supplementary Figure S1; Supplementary Table S5).

### Effect of blood counts on gestational duration

IVW analysis suggested a causal association between eosinophil count and gestational duration (*β*_IVW_ = −0.04 SD decrease in gestational duration per SD increase in eosinophil count, 95% CI: −0.07, −0.01, *p* = 0.006, P_het_ = 0.014), with the 95% confidence intervals around the causal effect estimates using the pleiotropy-robust methods overlapping with those from the IVW. No significant causal relationship was observed between any other blood count and gestational duration (Supplementary Figure S1; Supplementary Table S6).

### Effect of blood counts on pre-eclampsia and hypertensive disorders of pregnancy

Higher neutrophil counts were found to have a protective causal effect on the risk of pre-eclampsia and hypertensive disorders (OR 0.89, 95% CI: 0.82, 0.98, *p* = 0.012). Heterogeneity was again detected (P_het_ = 9.02 × 10^−7^). However, the 95% confidence intervals around the causal effect estimates using the pleiotropy-robust methods overlapped with those from the IVW but were wider. No other blood counts were found to have a causal relationship with pre-eclampsia and hypertensive disorders (Supplementary Figure S1; Supplementary Table S7).

### Two-step Mendelian randomisation and mediation analysis

In the two-step MR mediation analysis, the only combination that indicated a potential causal relationship between the exposure (BMI), the mediator (blood counts), and one of the pregnancy outcomes in the two-sample MR was between BMI, eosinophil count, and birth weight. Therefore, a formal two-step MR was performed only for this combination of traits.

**FIGURE 3:**
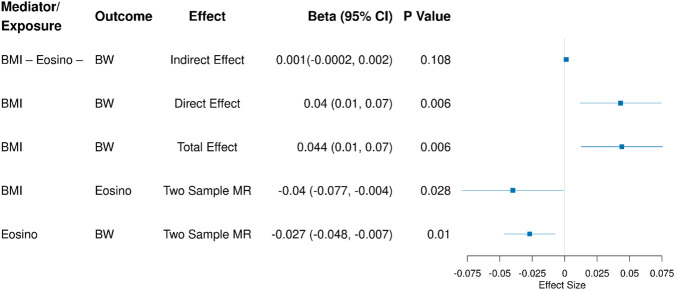
Mediation effects of eosinophil counts on the BMI–birth weight causal pathway. Eosino, eosinophil count; BMI, body mass index; BW, birth weight.

Using two-step MR, the total effect of BMI on birth weight was *β* = 0.04 SD increase in offspring birth weight per SD increase in maternal BMI (95% CI: 0.01, 0.07, *p* = 0.01). There was no evidence of an indirect effect (*β* = 0.001 SD increase in offspring birth weight per SD increase in maternal BMI, 95% CI: −0.0002, 0.002, *p* = 0.11). Subsequently, the direct effect of BMI on birth weight (*β* = 0.04 SD increase in offspring birth weight per SD increase in maternal BMI, 95% CI: 0.01, 0.07, *p* = 0.01) was very similar to the total effect (Figure 3).

## Discussion

We utilised genetic variants associated with BMI to investigate its relationship with pregnancy outcomes and determine whether this relationship is mediated by blood counts. We observed a causal relationship between maternal BMI and offspring birth weight, similar to but slightly lower than the findings of Tyrrell et al. (2016) (if one standard deviation in birth weight is equivalent to 492 g (Juliusdottir et al., 2021), then we estimate a 21.6 g [95% CI: 6.4, 36.4] increase in birth weight for each kg/m^2^ increase in maternal BMI, whereas Tyrrell et al. (2016) estimated a 55 g [95% CI: 17, 93] increase in birth weight). However, our sensitivity analyses indicate that no causal relationship exists between maternal BMI and birth weight after adjusting for horizontal pleiotropy. This could not be tested by Tyrrell et al. (2016) as they used a genetic score to test their causal relationship, which is not amenable to the pleiotropy-robust methods used in the current study. Notably, however, we identified a much stronger causal effect of maternal BMI on placental weight—an association that, to our knowledge, had not been previously assessed using MR. Given the established genetic relationships between birth weight, placental weight, and gestational duration, it is striking that we found no evidence for a causal relationship between maternal BMI and gestational duration. However, we successfully replicated the findings of Ardissino et al. (2024), confirming a causal relationship between maternal BMI and pre-eclampsia.

While observational studies have shown that maternal BMI is associated with both placental weight and birth weight independently (Macdonald et al., 2014) and that overweight and obese mothers tend to have disproportionately large placentas relative to birth weight (Richardson et al., 2017), our findings suggest a causal relationship more than five times stronger than its impact on birth weight. Placental function is shaped by maternal metabolic signals, many of which are altered by obesity. Emerging evidence suggests that obesity-driven placental changes mediate its adverse effects on fetal development (Jansson and Powell, 2013; Vaughan et al., 2017; Kelly et al., 2020). Maternal obesity-induced epigenetic modifications in the placenta, including altered DNA methylation and hydroxymethylation, are likely to drive placental overgrowth while impairing nutrient transfer efficiency, which may explain why maternal obesity has a greater effect on placental weight than on birth weight (Mitsuya et al., 2017; Johns et al., 2020).

Observational studies suggest that elevated BMI induces a pro-inflammatory state characterised by increased leukocyte levels. However, our MR analysis provides causal evidence that a higher BMI reduces several leukocyte and platelet counts. This replicates previously reported causal associations between maternal BMI and blood count traits (Thom et al., 2022) and suggests that alternative inflammatory mechanisms may be driving these effects.

Both eosinophil count and lymphocyte count were found to be negatively causally associated with birth weight, with eosinophil count also showing a negative causal effect on gestational duration. The maternal eosinophil count typically decreases slightly or remains stable throughout an uncomplicated pregnancy (Lurie et al., 2008; Abbassi-Ghanavati et al., 2009; Dockree et al., 2021). Nonetheless, several cytokines, particularly interleukin-5, can stimulate eosinophil production, leading to eosinophilia (Tefferi et al., 2006; Kanuru and Sapra, 2025). However, most cases of eosinophilia are hereditary, resulting from an autosomal dominant disorder located in the chromosomal region 5q31-q33 (Rioux et al., 1998). Elevated eosinophil counts are also associated with a severe form of asthma known as eosinophilic asthma, characterised by the TH2-high asthma endotype (Nelson et al., 2020). Maternal asthma is the most prevalent chronic condition during pregnancy and has been linked to reduced birth weight in offspring (Meakin et al., 2020; Lao and Annie Hui, 2022).

Lymphocyte counts are generally reported to decrease throughout pregnancy (Abbassi-Ghanavati et al., 2009; Dockree et al., 2021). Lymphocytes, specifically T cells and B cells, are crucial for balancing the maternal immune system during pregnancy to support fetal tolerance (Abu-Raya et al., 2020). Maternal lymphocytes, particularly decidual natural killer (dNK) cells, are pivotal in the formation of the decidua and the success of implantation (Koopman et al., 2003; Ander et al., 2019). While dNK cells are less cytotoxic, other subsets, such as peripheral and endometrial natural killer cells, tend to be more cytotoxic; accordingly, elevated endometrial NK cells have been associated with recurrent pregnancy loss and recurrent implantation failure (Braun et al., 2023; Cuadrado-Torroglosa et al., 2024). This suggests that an imbalance in the maternal immune system lymphocytes negatively affects the maternal revascularisation of maternal spiral arteries and fetal trophoblast invasion of the endometrium (Braun et al., 2023; Cuadrado-Torroglosa et al., 2024), resulting in fetal growth restriction or lower birth weight. While elevated lymphocyte counts during pregnancy often result from infection or chronic inflammation, there is also a substantial genetic contribution to an individual’s lymphocyte count (Evans et al., 1999).

This study found that higher neutrophil counts were associated with a lower risk of pre-eclampsia and hypertensive disorders of pregnancy. Although this may seem counterintuitive given the inflammatory nature of pre-eclampsia, evidence suggests that neutrophils play a regulatory role in immune balance rather than solely driving inflammation (Bert et al., 2021; Chan et al., 2022). Specifically, a distinction must be made between neutrophil quantity and quality; while hyper-activated neutrophils, often driven by inflammatory placental factors such as IL-32β, induce vascular damage, a robust pool of “resting” neutrophils may be essential for early pregnancy processes such as placental vascularisation and maintaining immune tolerance (Liu et al., 2021). A more robust immune response may help mitigate excessive inflammation in pregnancy, potentially explaining our findings. This “two-hit” hypothesis suggests that an adequate baseline neutrophil count provides a protective buffer, preventing early placental stress that would otherwise trigger a pathological inflammatory cascade. A MR study by Zeng et al. (2024), investigating immune dysregulation and inflammatory biomarkers in pre-eclampsia, found no association between genetically predicted neutrophil count and pre-eclampsia (Zeng et al., 2024). However, our study had a larger sample size and a broader phenotype definition, including pre-eclampsia and hypertensive disorders of pregnancy, which increased sensitivity and may have improved our ability to detect an association. One limitation is that the Tyrmi et al. (2023) GWAS uses a broad ‘hypertensive disorders of pregnancy’ phenotype, which is naturally weighted toward late-onset cases driven by maternal cardiovascular risk. However, immune-mediated pathology and abnormal placentation are primary drivers of early-onset pre-eclampsia (Staff, 2019). Since neutrophils are specifically involved in early placental vascularisation (Liu et al., 2021), using a mixed-phenotype outcome likely dilutes these subtype-specific immune signals. Our estimates should, therefore, be viewed as a general genetic liability across heterogeneous phenotypes, which may underestimate the specific protective role of neutrophils in the immune-driven early-onset subtype.

Two-step MR analyses found no evidence that maternal blood counts mediate the effect of maternal BMI on offspring birth weight. This suggests that any inflammatory response linking maternal BMI to birth weight, placental weight, and pre-eclampsia operates independently of the blood count-related inflammatory pathways, which themselves are causally associated with birth weight, gestational duration, and pre-eclampsia. These results underscore a complex interplay: although maternal BMI is causally associated with changes in several blood counts, these alterations do not explain its overall effect on offspring birth weight, placental weight, or pre-eclampsia, indicating that other inflammatory pathways are likely involved.

The strengths of our study include access to large GWASs for all our traits and the partitioned maternal and fetal genetic effects of our pregnancy outcomes. Furthermore, we used several sensitivity analyses with differing assumptions regarding horizontal pleiotropy, which strengthens confidence in our findings. Conversely, the study also has limitations. Horizontal pleiotropy in MR can only be partially accounted for by methods such as median- and mode-based MR and MR-Egger. The more complex a disease and the more variants used to investigate the causal relationship, the more likely the SNP-outcome relationship is affected by unknown horizontal pleiotropic pathways (Bowden et al., 2018). Second, the lack of population diversity in the selected GWASs may limit the generalisable of our findings to populations outside the European cohorts. Thus, further research is required in other ancestries before conclusions can be drawn regarding the complex relationship between BMI, immune cell counts, and pregnancy outcomes. Third, we used a nominal *p*-value threshold (*p* < 0.05) to select causal relationships for inclusion in the two-step MR analysis. We acknowledge that this is a liberal threshold, but it identified only one combination of traits to be formally tested for mediation. Nevertheless, the relationships between BMI, eosinophil count, and birth weight should be replicated in an independent study. Fourth, there is limited statistical power to detect mediation using two-step MR. Although a formal mediation analysis could be conducted, for example, the BMI–eosinophil count–birth weight pathway, the individual causal effects at each step were modest. Because the indirect effect in two-step MR is estimated as the product of two causal effects, even relatively weak associations substantially reduce power to detect mediation. This limitation was further compounded by our requirement for evidence of causal relationships at each step prior to formal mediation analysis. Consequently, the absence of a statistically significant indirect effect should not be interpreted as strong evidence against mediation, but rather reflects limited power to detect small mediation effects. Finally, the genetic instruments for immune cell counts were derived from predominantly non-pregnant populations. Because pregnancy involves substantial immunological adaptation that may alter SNP–immune cell associations, the assumption that these relationships are comparable during pregnancy represents a limitation; pregnancy-specific GWAS will be needed to confirm these mediating pathways.

## Conclusion

This study demonstrates that elevated maternal BMI has a causal influence on placental weight and that the previously identified relationship with birth weight may be substantially affected by pleiotropy Additionally, these causal relationships are not mediated via maternal blood counts, indicating that intervening on blood counts during pregnancy is unlikely to influence fetal or placental growth. Continuing to explore the genetic factors, immune cell function, and environmental influences on pregnancy outcomes will be crucial in advancing our knowledge and enhancing maternal and child health.

## Data Availability

The original contributions presented in the study are included in the article/Supplementary Material, further inquiries can be directed to the corresponding authors.
