# Supplementary material for: Causal effects of maternal BMI on pregnancy outcomes: a Mendelian randomisation study investigating the mediating role of blood counts
Source: Front Genet. 2026 Apr 24;17:1697926. doi: 10.3389/fgene.2026.1697926 (PMC13152253; doi:10.3389/fgene.2026.1697926)
Supplement: Supplementary file 1 [file Supplementaryfile1.docx]

**Causal Effects of Maternal BMI on Pregnancy Outcomes: A Mendelian Randomisation Study on the Mediating Role of Blood Counts**

**Supplementary Methods**

**Weighted Linear Model to Partition the SNP Effect Estimates on Birth Weight.**

The full derivation of the weighted linear model can be found in Warrington *et al* [[1]](https://paperpile.com/c/4KLcVF/rWNak). Here we summarise the relevant parts that were required for this current study.

To derive the adjusted maternal and fetal effect size estimates on birth weight, we used the following equations:

[
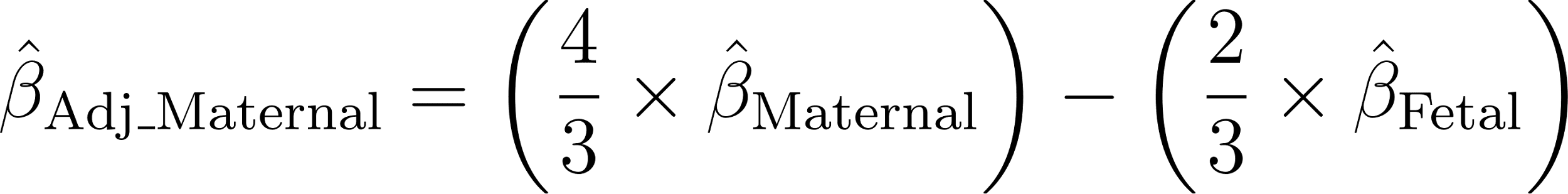
](https://www.codecogs.com/eqnedit.php?latex=%20%5Chat%7B%5Cbeta%7D_%7B%5Ctext%7BAdj%5C_Maternal%7D%7D%20%3D%20%5Cleft(%20%5Cfrac%7B4%7D%7B3%7D%20%5Ctimes%20%5Chat%7B%5Cbeta%7D_%7B%5Ctext%7BMaternal%7D%7D%20%5Cright)%20-%20%5Cleft(%20%5Cfrac%7B2%7D%7B3%7D%20%5Ctimes%20%5Chat%7B%5Cbeta%7D_%7B%5Ctext%7BFetal%7D%7D%20%5Cright)%20#0)

[
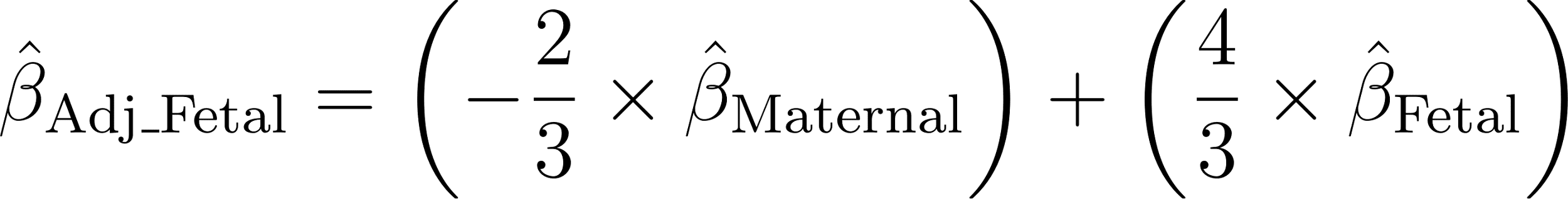
](https://www.codecogs.com/eqnedit.php?latex=%20%5Chat%7B%5Cbeta%7D_%7B%5Ctext%7BAdj%5C_Fetal%7D%7D%20%3D%20%5Cleft(%20-%5Cfrac%7B2%7D%7B3%7D%20%5Ctimes%20%5Chat%7B%5Cbeta%7D_%7B%5Ctext%7BMaternal%7D%7D%20%5Cright)%20%2B%20%5Cleft(%20%5Cfrac%7B4%7D%7B3%7D%20%5Ctimes%20%5Chat%7B%5Cbeta%7D_%7B%5Ctext%7BFetal%7D%7D%20%5Cright)%20#0)

Where [
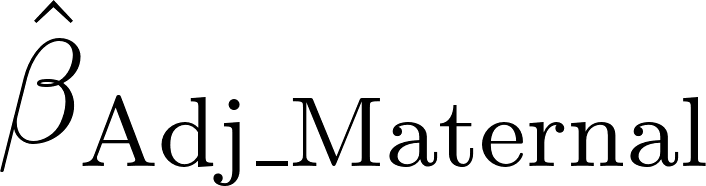
](https://www.codecogs.com/eqnedit.php?latex=%5Chat%7B%5Cbeta%7D_%7B%5Ctext%7BAdj%5C_Maternal%7D%7D#0) is the estimated effect size of maternal genotype on offspring birth weight after adjusting for fetal genotype, [
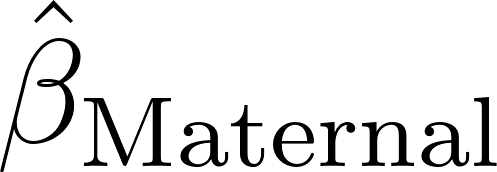
](https://www.codecogs.com/eqnedit.php?latex=%5Chat%7B%5Cbeta%7D_%7B%5Ctext%7BMaternal%7D%7D#0) is the unadjusted estimated effect size of maternal genotype on offspring birth weight from the DECODE GWAS, [
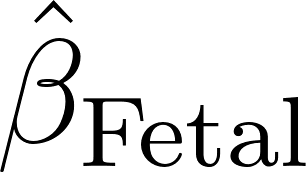
](https://www.codecogs.com/eqnedit.php?latex=%20%5Chat%7B%5Cbeta%7D_%7B%5Ctext%7BFetal%7D%7D#0) is the unadjusted estimated effect size of fetal genotype on their own birth weight from the DECODE GWAS and [
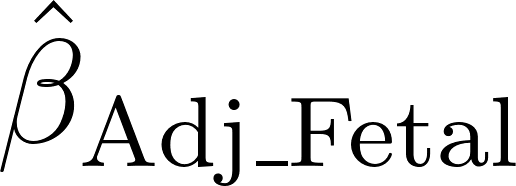
](https://www.codecogs.com/eqnedit.php?latex=%5Chat%7B%5Cbeta%7D_%7B%5Ctext%7BAdj%5C_Fetal%7D%7D#0) is the estimated effect size of fetal genotype on their own birth weight after adjusting for maternal genotype.

As no standard error is reported in the maternal or fetal GWAS summary statistics for birth weight, we derive them from the P-values. First, find the Z statistic for each:

[
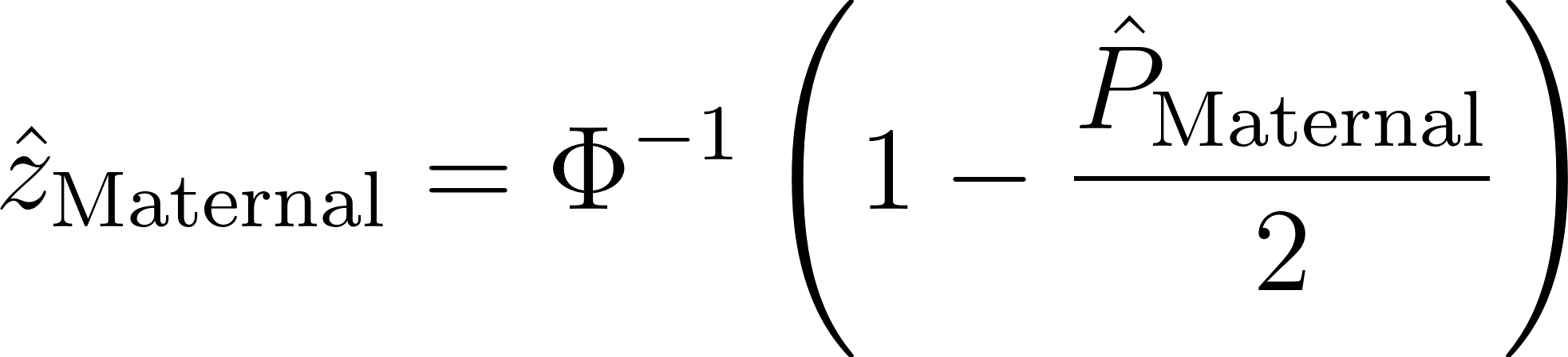
](https://www.codecogs.com/eqnedit.php?latex=%20%5Chat%7Bz%7D_%7B%5Ctext%7BMaternal%7D%7D%20%3D%20%5CPhi%5E%7B-1%7D%5Cleft(1%20-%20%5Cfrac%7B%5Chat%7BP%7D_%7B%5Ctext%7BMaternal%7D%7D%7D%7B2%7D%5Cright)%20#0)

[
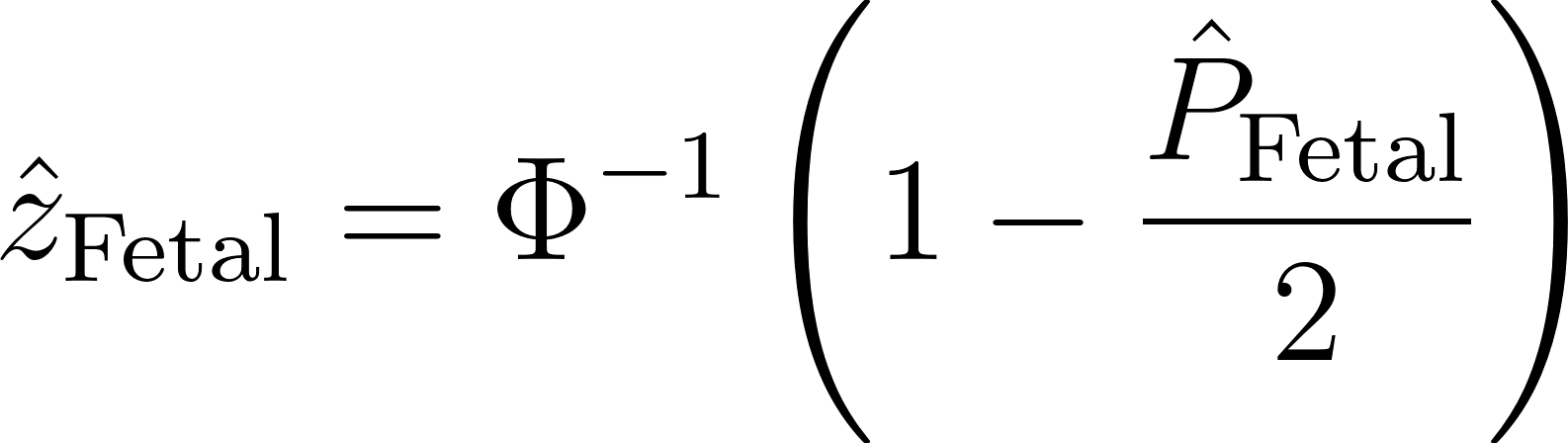
](https://www.codecogs.com/eqnedit.php?latex=%20%5Chat%7Bz%7D_%7B%5Ctext%7BFetal%7D%7D%20%3D%20%5CPhi%5E%7B-1%7D%5Cleft(1%20-%20%5Cfrac%7B%5Chat%7BP%7D_%7B%5Ctext%7BFetal%7D%7D%7D%7B2%7D%5Cright)%20#0)

Where [
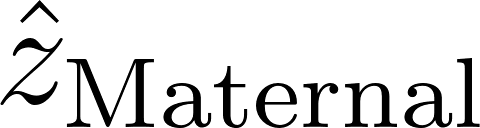
](https://www.codecogs.com/eqnedit.php?latex=%20%5Chat%7Bz%7D_%7B%5Ctext%7BMaternal%7D%7D#0) is the derived Z statistic for the maternal estimate and [
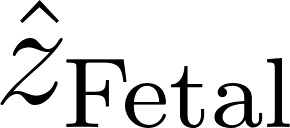
](https://www.codecogs.com/eqnedit.php?latex=%20%5Chat%7Bz%7D_%7B%5Ctext%7BFetal%7D%7D#0) is the derived Z statistic for the fetal estimate.

Φ denotes the cumulative distribution function (CDF) of the standard normal distribution.

From this, we calculate the standard error of the (unadjusted) SNP effects of maternal SNP on offspring birth weight ([
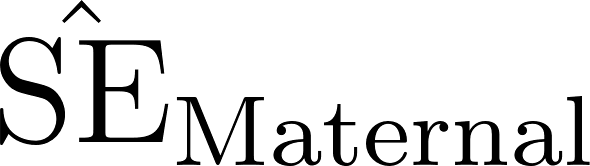
](https://www.codecogs.com/eqnedit.php?latex=%20%5Chat%7B%5Ctext%7BSE%7D%7D_%7B%5Ctext%7BMaternal%7D%7D#0)) and fetal SNP on their own birth weight ([
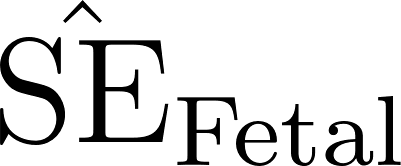
](https://www.codecogs.com/eqnedit.php?latex=%20%5Chat%7B%5Ctext%7BSE%7D%7D_%7B%5Ctext%7BFetal%7D%7D#0) ) using the following:

[
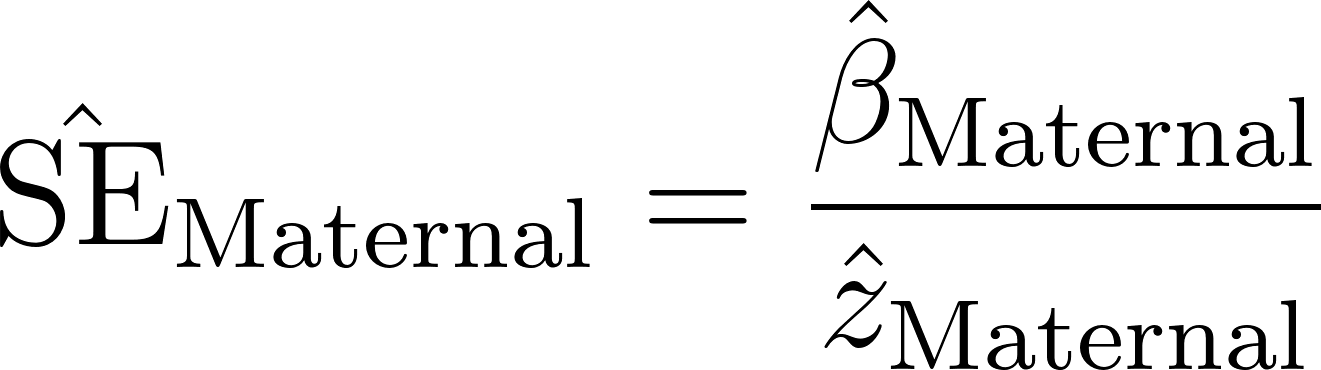
](https://www.codecogs.com/eqnedit.php?latex=%20%5Chat%7B%5Ctext%7BSE%7D%7D_%7B%5Ctext%7BMaternal%7D%7D%20%3D%20%5Cfrac%7B%5Chat%7B%5Cbeta%7D_%7B%5Ctext%7BMaternal%7D%7D%7D%7B%5Chat%7Bz%7D_%7B%5Ctext%7BMaternal%7D%7D%7D%20#0)

[
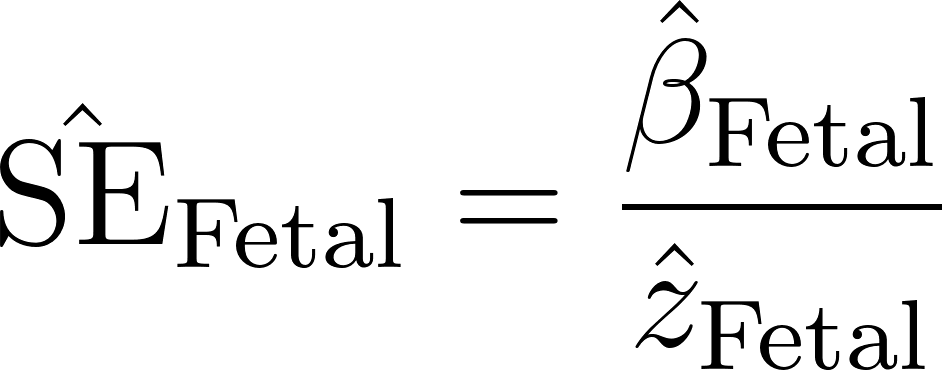
](https://www.codecogs.com/eqnedit.php?latex=%20%5Chat%7B%5Ctext%7BSE%7D%7D_%7B%5Ctext%7BFetal%7D%7D%20%3D%20%5Cfrac%7B%5Chat%7B%5Cbeta%7D_%7B%5Ctext%7BFetal%7D%7D%7D%7B%5Chat%7Bz%7D_%7B%5Ctext%7BFetal%7D%7D%7D%20#0)

Finally, we use these in the following equation to calculate the standard error for the adjusted maternal SNP effect on offspring birth weight ([
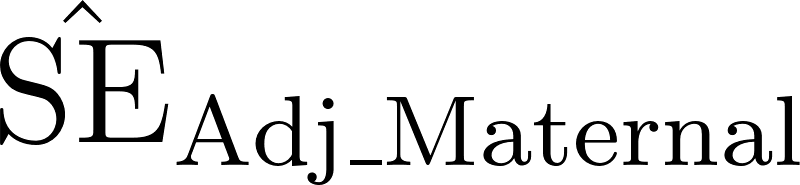
](https://www.codecogs.com/eqnedit.php?latex=%5Chat%7B%5Ctext%7BSE%7D%7D_%7B%5Ctext%7BAdj%5C_Maternal%7D%7D#0)):

[
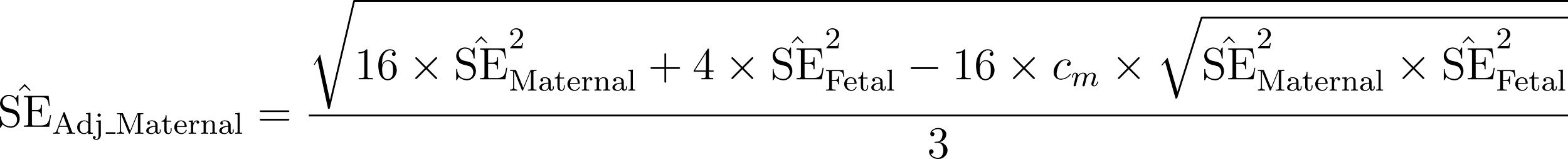
](https://www.codecogs.com/eqnedit.php?latex=%20%5Chat%7B%5Ctext%7BSE%7D%7D_%7B%5Ctext%7BAdj%5C_Maternal%7D%7D%20%3D%20%5Cfrac%7B%5Csqrt%7B16%20%5Ctimes%20%5Chat%7B%5Ctext%7BSE%7D%7D_%7B%5Ctext%7BMaternal%7D%7D%5E2%20%2B%204%20%5Ctimes%20%5Chat%7B%5Ctext%7BSE%7D%7D_%7B%5Ctext%7BFetal%7D%7D%5E2%20-%2016%20%5Ctimes%20c_m%20%5Ctimes%20%5Csqrt%7B%5Chat%7B%5Ctext%7BSE%7D%7D_%7B%5Ctext%7BMaternal%7D%7D%5E2%20%5Ctimes%20%5Chat%7B%5Ctext%7BSE%7D%7D_%7B%5Ctext%7BFetal%7D%7D%5E2%7D%7D%7D%7B3%7D%20#0)

Where c_m_ is the genetic covariance between the maternal and fetal effects.

Then the subsequent Z-score to calculate the adjusted P-value:

[
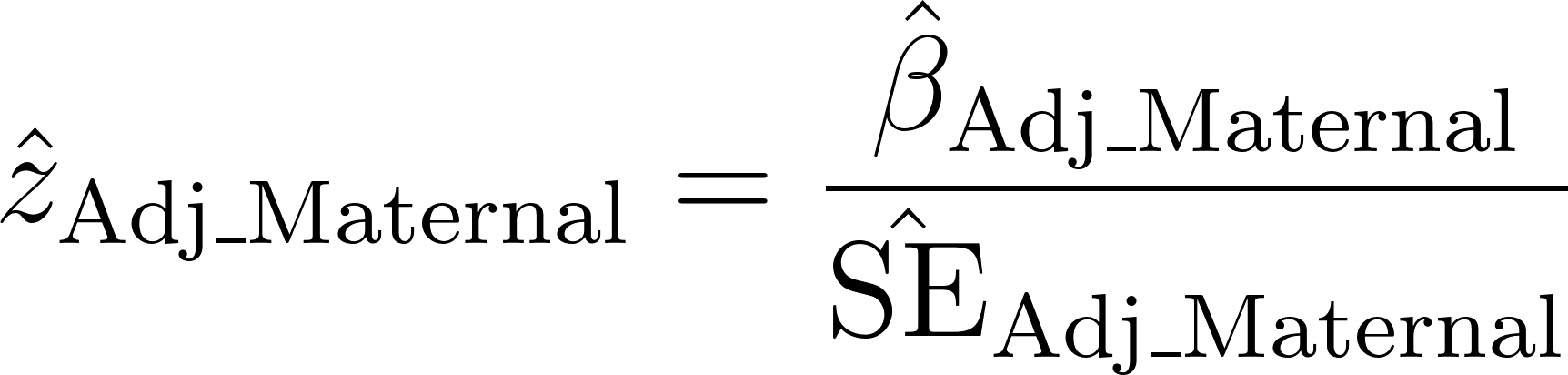
](https://www.codecogs.com/eqnedit.php?latex=%20%5Chat%7Bz%7D_%7B%5Ctext%7BAdj%5C_Maternal%7D%7D%20%3D%20%5Cfrac%7B%5Chat%7B%5Cbeta%7D_%7B%5Ctext%7BAdj%5C_Maternal%7D%7D%7D%7B%5Chat%7B%5Ctext%7BSE%7D%7D_%7B%5Ctext%7BAdj%5C_Maternal%7D%7D%7D%20#0)

[
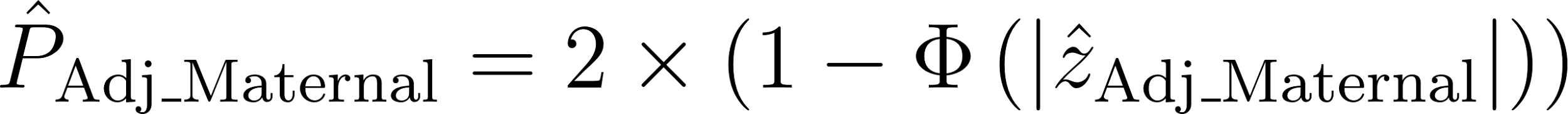
](https://www.codecogs.com/eqnedit.php?latex=%20%5Chat%7BP%7D_%7B%5Ctext%7BAdj%5C_Maternal%7D%7D%20%3D%202%20%5Ctimes%20%5Cleft(%201%20-%20%5CPhi%20%5Cleft(%20%5Cleft%7C%20%5Chat%7Bz%7D_%7B%5Ctext%7BAdj%5C_Maternal%7D%7D%20%5Cright%7C%20%5Cright)%20%5Cright)%20#0)

**Supplementary Figure 1: Results from Two-Sample Mendelian Randomisation.** BMI: Body Mass Index, IVW: Inverse Variance Weighted, CI: Confidence Interval
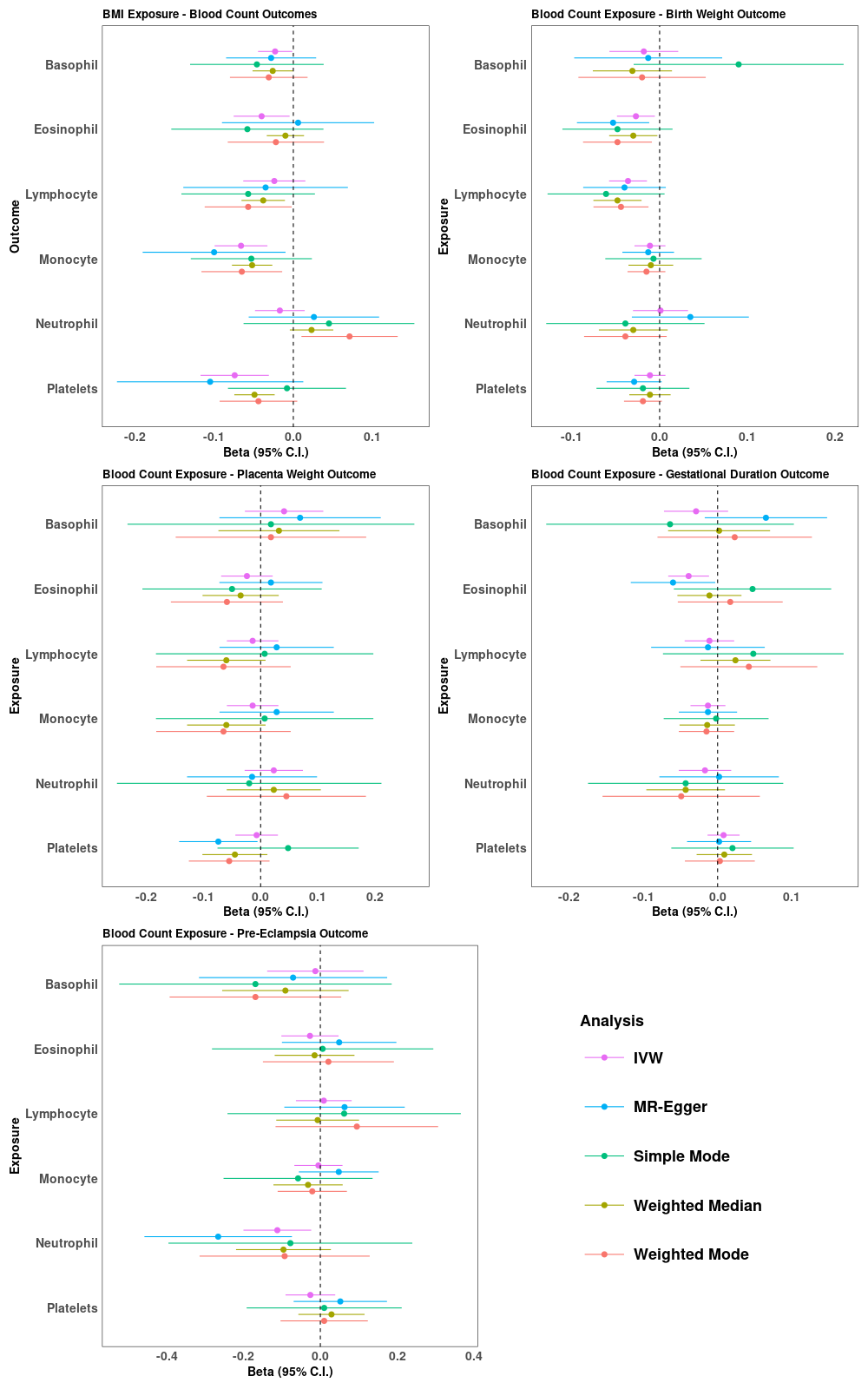


**Supplementary Table 1: Number of variants for each two-sample Mendelian randomisation and corresponding F-statistics.**

| **Instrumental Variable** | **Outcome** | **Number of SNPs** | **Mean F-Statistic** |
| --- | --- | --- | --- |
| BMI | Birth Weight | 504 | 72.3 |
| Basophil | Birth Weight | 163 | 93.0 |
| Eosinophil | Birth Weight | 390 | 118.5 |
| Lymphocytes | Birth Weight | 432 | 101.0 |
| Monocytes | Birth Weight | 444 | 150.8 |
| Neutrophils | Birth Weight | 336 | 87.6 |
| Platelets | Birth Weight | 497 | 136.5 |
| BMI | Placental Weight | 521 | 72.0 |
| Basophil | Placental Weight | 155 | 94.8 |
| Eosinophil | Placental Weight | 360 | 122.8 |
| Lymphocytes | Placental Weight | 402 | 99.8 |
| Monocytes | Placental Weight | 418 | 157.0 |
| Neutrophils | Placental Weight | 312 | 89.1 |
| Platelets | Placental Weight | 473 | 139.2 |
| BMI | Gestational Duration | 475 | 73.2 |
| Basophil | Gestational Duration | 136 | 97.6 |
| Eosinophil | Gestational Duration | 317 | 121.3 |
| Lymphocytes | Gestational Duration | 346 | 96.1 |
| Monocytes | Gestational Duration | 366 | 160.9 |
| Neutrophils | Gestational Duration | 275 | 86.3 |
| Platelets | Gestational Duration | 418 | 140.0 |
| BMI | Pre-eclampsia | 511 | 71.8 |
| Basophil | Pre-eclampsia | 153 | 94.1 |
| Eosinophil | Pre-eclampsia | 365 | 117.0 |
| Lymphocytes | Pre-eclampsia | 404 | 98.3 |
| Monocytes | Pre-eclampsia | 417 | 153.7 |
| Neutrophils | Pre-eclampsia | 317 | 88.8 |
| Platelets | Pre-eclampsia | 472 | 137.7 |

#BMI: body mass index, Pre-eclampsia: Pre-eclampsia and hypertensive disorders of pregnancy

**Supplementary Table 2: Two-Sample Mendelian Randomisation of Body Mass Index (Exposure) on Pregnancy Outcomes (Outcome).**

| **Outcome** | **Method** | **Analysis** | | | | | |
| --- | --- | --- | --- | --- | --- | --- | --- |
|  |  | **IVW** | **Simple Mode** | **Weighted Mode** | **Weighted Median** | **MR-Egger** | **MR-Egger Intercept** |
| Birth Weight | Beta | 0.044 | -0.021 | -0.042 | 0.000 | -0.029 | 0.001 |
|  | 95% C.I. | 0.013, 0.074 | -0.146, 0.104 | -0.093, 0.009 | -0.035, 0.035 | -0.113, 0.055 | -0.001, 0.003 |
|  | P Value | 0.005 | 0.746 | 0.107 | 1.000 | 0.489 | 0.065 |
|  | Heterogeneity P | 2.79E-68 |  |  |  | 2.38E-67 |  |
|  | N Snps | 504 |  | F-Statistic | 72.3 |  |  |
| Placental Weight | Beta | 0.164 | 0.215 | 0.305 | 0.234 | 0.355 | -0.003 |
|  | 95% C.I. | 0.101, 0.227 | -0.079, 0.509 | 0.144, 0.466 | 0.144, 0.324 | 0.186, 0.524 | -0.005,  -0.001 |
|  | P Value | 2.92e-07 | 0.150 | 2.09e-04 | 4.81e-07 | 4.13e-05 | 0.017 |
|  | Heterogeneity P | 7.47E-07 |  |  |  | 1.68E-06 |  |
|  | N Snps | 521 |  | F-Statistic | 72.03 |  |  |
| Gestational Duration | Beta | 0.008 | 0.107 | 0.084 | 0.048 | 0.073 | -0.001 |
|  | 95% C.I. | -0.027, 0.043 | -0.064, 0.278 | -0.038, 0.206 | -0.009, 0105 | -0.023, 0.169 | -0.003, 0.001 |
|  | P Value | 0.674 | 0.222 | 0.173 | 0.098 | 0.136 | 0.151 |
|  | Heterogeneity P | 0.028 |  |  |  | 0.030 |  |
|  | N Snps | 475 |  | F-Statistic | 73.2 |  |  |
| Pre-eclampsia and Hypertensive Disorders of Pregnancy | Odds Ratio | 1.747 | 2.145 | 2.192 | 1.917 | 2.232 | 0.996 |
|  | 95% C.I. | 1.587, 1.923 | 1.327, 3.467 | 1.663, 2.890 | 1.665, 2.208 | 1.723, 2.891 | 0.992, 1.000 |
|  | P Value | 6.33E-30 | 0.002 | 4.34E-08 | 8.98E-20 | 2.03E-09 | 0.045 |
|  | Heterogeneity P | 1.18E-06 |  |  |  | 2.00E-06 |  |
|  | N Snps | 511 |  | F-Statistic | 71.8 |  |  |

#IVW: Inverse Variance Weighted, C.I.: Confidence Interval

**Supplementary Table 3: Two-Sample Mendelian Randomisation of Body Mass Index (Exposure) on Blood Counts (Outcome).**

| **Outcome** | **Method** | **Analysis** | | | | | |
| --- | --- | --- | --- | --- | --- | --- | --- |
|  |  | **IVW** | **Simple Mode** | **Weighted Mode** | **Weighted Median** | **MR-Egger** | **MR-Egger Int** |
| Basophil | Beta | -0.023 | -0.046 | -0.031 | -0.026 | -0.028 | 7.96E-05 |
|  | 95% C.I. | -0.045,  -0.002 | -0.130, 0.038 | -0.080, 0.018 | -0.051,  -0.001 | -0.085, 0.029 | -0.001, 0.001 |
|  | P Value | 0.029 | 0.291 | 0.210 | 0.052 | 0.333 | 0.864 |
|  | Heterogeneity P | 6.46E-69 |  |  |  | 4.16E-69 |  |
|  | N Snps | 521 |  | F-Statistic | 72.0 |  |  |
| Eosinophil | Beta | -0.040 | -0.058 | -0.022 | -0.010 | 0.006 | -0.001 |
|  | 95% C.I. | -0.075,  -0.004 | -0.154, 0.038 | -0.083, 0.039 | -0.034,  0.014 | -0.090, 0.102 | -0.003, 0.001 |
|  | P Value | 0.028 | 0.234 | 0.483 | 0.426 | 0.896 | 0.308 |
|  | Heterogeneity P | 0 |  |  |  | 0 |  |
|  | N Snps | 521 |  | F-Statistic | 72.0 |  |  |
| Lymphocytes | Beta | -0.024 | -0.057 | -0.057 | -0.038 | -0.035 | 1.97E-04 |
|  | 95% C.I. | -0.063, 0.015 | -0.141, 0.027 | -0.112,  -0.002 | -0.065,  -0.011 | -0.139, 0.069 | -0.002, 0.002 |
|  | P Value | 0.225 | 0.191 | 0.046 | 0.007 | 0.504 | 0.815 |
|  | Heterogeneity P | 0 |  |  |  | 0 |  |
|  | N Snps | 521 |  | F-Statistic | 72.0 |  |  |
| Monocytes | Beta | -0.066 | -0.053 | -0.065 | -0.052 | -0.100 | 0.001 |
|  | 95% C.I. | -0.099,  -0.033 | -0.129, 0.023 | -0.116,  -0.014 | -0.077,  -0.027 | -0.190,  -0.010 | -0.001, 0.003 |
|  | P Value | 9.80E-05 | 0.172 | 0.012 | 9.23E-05 | 0.028 | 0.419 |
|  | Heterogeneity P | 0 |  |  |  | 0 |  |
|  | N Snps | 521 |  | F-Statistic | 72.0 |  |  |
| Neutrophils | Beta | -0.017 | 0.045 | 0.071 | 0.023 | 0.026 | -0.001 |
|  | 95% C.I. | -0.048, 0.014 | -0.063, 0.153 | 0.010, 0.132 | -0.004,  0.050 | -0056, 0.108 | -0.003, 0.001 |
|  | P Value | 0.296 | 0.417 | 0.021 | 0.095 | 0.540 | 0.281 |
|  | Heterogeneity P | 0 |  |  |  | 0 |  |
|  | N Snps | 521 |  | F-Statistic | 72.0 |  |  |
| Platelets | Beta | -0.074 | -0.008 | -0.044 | -0.049 | -0.105 | 0.001 |
|  | 95% C.I. | -0.117,  -0.031 | -0.082, 0.066 | -0.093, 0.005 | -0.074,  -0.024 | -0.223, 0.013 | -0.001, 0.003 |
|  | P Value | 0.001 | 0.826 | 0.082 | 8.37E-05 | 0.080 | 0.577 |
|  | Heterogeneity P | 0 |  |  |  | 0 |  |
|  | N Snps | 521 |  | F-Statistic | 72.0 |  |  |

#IVW: Inverse Variance Weighted, C.I.: Confidence Interval, MR-Egger Int: MR-Egger Intercept

**Supplementary Table 4: Two-Sample Mendelian Randomisation of Blood Counts (Exposure) and Birth Weight (Outcome).**

| **Exposure** | **Method** | **Analysis** | | | | | |
| --- | --- | --- | --- | --- | --- | --- | --- |
|  |  | **IVW** | **Simple Mode** | **Weighted Mode** | **Weighted Median** | **MR-Egger** | **MR-Egger Int** |
| Basophil | Beta | -0.018 | 0.090 | -0.020 | -0.031 | -0.013 | -0.0002 |
|  | 95% C.I. | -0.057, 0.021 | -0.030, 0.210 | -0.092, 0.053 | -0.076,  0.014 | -0.097, 0.071 | -0.002, 0.002 |
|  | P Value | 0.364 | 0.144 | 0.599 | 0.184 | 0.767 | 0.885 |
|  | Heterogeneity P | 5.05e-20 |  |  |  | 3.30e-20 |  |
|  | N Snps | 163 |  | F-Statistic | 93.0 |  |  |
| Eosinophil | Beta | -0.027 | -0.048 | -0.048 | -0.030 | -0.053 | 0.001 |
|  | 95% C.I. | -0.049,  -0.005 | -0.111, 0.015 | -0.087,  -0.009 | -0.057,  -0.003 | -0.094,  -0.012 | -0.001, 0.003 |
|  | P Value | 0.010 | 0.130 | 0.016 | 0.034 | 0.014 | 0.169 |
|  | Heterogeneity P | 5.62e-30 |  |  |  | 1.06e-29 |  |
|  | N Snps | 390 |  | F-Statistic | 118.5 |  |  |
| Lymphocytes | Beta | -0.036 | -0.061 | -0.044 | -0.048 | -0.040 | 0.0001 |
|  | 95% C.I. | -0.058,  -0015 | -0.128, 0.006 | -0.075,  -0.013 | -0.075,  -0.021 | -0.087, 0.007 | -0.002, 0.002 |
|  | P Value | 0.001 | 0.072 | 0.008 | 0.001 | 0.091 | 0.851 |
|  | Heterogeneity P | 4.47e-35 |  |  |  | 3.14e-35 |  |
|  | N Snps | 432 |  | F-Statistic | 101.0 |  |  |
| Monocytes | Beta | -0.011 | -0.007 | -0.015 | -0.010 | -0.013 | 9.24e-05 |
|  | 95% C.I. | -0.029, 0.007 | -0.062, 0.048 | -0.037, 0.007 | -0.035,  0.015 | -0.042, 0.016 | -0.001, 0.001 |
|  | P Value | 0.223 | 0.795 | 0.195 | 0.403 | 0.374 | 0.841 |
|  | Heterogeneity P | 6.08e-33 |  |  |  | 4.35e-33 |  |
|  | N Snps | 444 |  | F-Statistic | 150.8 |  |  |
| Neutrophils | Beta | 0.001 | -0.039 | -0.039 | -0.030 | 0.035 | -0.001 |
|  | 95% C.I. | -0.030, 0.032 | -0.129, 0.051 | -0.086, 0.008 | -0.069,  0.009 | -0.032, 0.102 | -0.003, 0.001 |
|  | P Value | 0.974 | 0.397 | 0.103 | 0.118 | 0.299 | 0.250 |
|  | Heterogeneity P | 4.97e-46 |  |  |  | 8.65e-46 |  |
|  | N Snps | 336 |  | F-Statistic | 87.6 |  |  |
| Platelets | Beta | -0.011 | -0.019 | -0.019 | -0.011 | -0.029 | 0.001 |
|  | 95% C.I. | -0.029, 0.007 | -0.072, 0.034 | -0.041, 0.003 | -0.035,  0.013 | -0.060, 0.002 | 2.00E-05, 0.002 |
|  | P Value | 0.211 | 0.487 | 0.094 | 0.333 | 0.068 | 0.172 |
|  | Heterogeneity P | 9.40e-33 |  |  |  | 1.64e-32 |  |
|  | N Snps | 497 |  | F-Statistic |  |  |  |

#IVW: Inverse Variance Weighted, C.I.: Confidence Interval, MR-Egger Int: MR-Egger Intercept

**Supplementary Table 5: Two-Sample Mendelian Randomisation of Blood Counts (Exposure) and Placental Weight (Outcome).**

| **Exposure** | **Method** | **Analysis** | | | | | |
| --- | --- | --- | --- | --- | --- | --- | --- |
|  |  | **IVW** | **Simple Mode** | **Weighted Mode** | **Weighted Median** | **MR-Egger** | **MR-Egger Int** |
| Basophil | Beta | 0.041 | 0.018 | 0.018 | 0.032 | 0.069 | -0.001 |
|  | 95% C.I. | -0.028, 0.110 | -0.233, 0.269 | -0.149, 0.185 | -0.074, 0.138 | -0.072, 0.210 | -0.005, 0.003 |
|  | P Value | 0.237 | 0.890 | 0.836 | 0.554 | 0.336 | 0.657 |
|  | Heterogeneity P | 0.351 |  |  |  |  | 0.334 |
|  | N Snps | 155 |  | F-Statistic | 94.8 |  |  |
| Eosinophil | Beta | -0.024 | -0.050 | -0.059 | -0.035 | 0.018 | -0.001 |
|  | 95% C.I. | -0.070, 0.021 | -0.207, 0.107 | -0.157, 0.039 | -0.102, 0.032 | -0.072, 0.108 | -0.003, 0.001 |
|  | P Value | 0.290 | 0.536 | 0.238 | 0.303 | 0.694 | 0.290 |
|  | Heterogeneity P | 0.003 |  |  |  | 0.003 |  |
|  | N Snps | 360 |  | F-Statistic | 122.8 |  |  |
| Lymphocytes | Beta | -0.014 | 0.007 | -0.065 | -0.060 | 0.028 | -0.001 |
|  | 95% C.I. | -0.060, 0.031 | -0.183, 0.197 | -0.183, 0.053 | -0.129, 0.009 | -0.072, 0.128 | -0.003, 0.001 |
|  | P Value | 0.536 | 0.944 | 0.280 | 0.091 | 0.590 | 0.362 |
|  | Heterogeneity P | 0.114 |  |  |  | 0.113 |  |
|  | N Snps | 402 |  | F-Statistic | 99.8 |  |  |
| Monocytes | Beta | 0.009 | -0.057 | 0.010 | 0.014 | 0.032 | -0.001 |
|  | 95% C.I. | -0.026, 0.044 | -0.182, 0.068 | -0.053, 0.073 | -0.045, 0.073 | -0.029, 0.093 | -0.003, 0.001 |
|  | P Value | 0.643 | 0.370 | 0.762 | 0.651 | 0.294 | 0.335 |
|  | Heterogeneity P | 0.021 |  |  |  | 0.021 |  |
|  | N Snps | 418 |  | F-Statistic | 157.0 |  |  |
| Neutrophils | Beta | 0.023 | -0.020 | 0.045 | 0.023 | -0.015 | 0.001 |
|  | 95% C.I. | -0.028, 0.074 | -0.251, 0.211 | -0.094, 0.184 | -0.059, 0.105 | -0.129, 0.099 | -0.001, 0.003 |
|  | P Value | 0.377 | 0.862 | 0.527 | 0.586 | 0.792 | 0.461 |
|  | Heterogeneity P | 0.377 |  |  |  | 0.371 |  |
|  | N Snps | 312 |  | F-Statistic | 89.1 |  |  |
| Platelets | Beta | -0.007 | 0.048 | -0.055 | -0.045 | -0.074 | 0.002 |
|  | 95% C.I. | -0.044, 0.030 | -0.075, 0.171 | -0.126, 0.016 | -0.102, 0.012 | -0.143,  -0.005 | 4.00e-5, 0.004 |
|  | P Value | 0.711 | 0.450 | 0.129 | 0.126 | 0.037 | 0.025 |
|  | Heterogeneity P | 0.001 |  |  |  | 0.001 |  |
|  | N Snps | 473 |  | F-Statistic | 139.2 |  |  |

#IVW: Inverse Variance Weighted, C.I.: Confidence Interval MR-Egger Int: MR-Egger Intercept

**Supplementary Table 6: Two-Sample Mendelian Randomisation of Blood Counts (Exposure) and Gestational Duration (Outcome).**

| **Exposure** | **Method** | **Analysis** | | | | | |
| --- | --- | --- | --- | --- | --- | --- | --- |
|  |  | **IVW** | **Simple Mode** | **Weighted Mode** | **Weighted Median** | **MR-Egger** | **MR-Egger Int** |
| Basophil | Beta | -0.029 | -0.064 | 0.023 | 0.002 | 0.065 | -0.003 |
|  | 95% C.I. | -0.072, 0.014 | -0.231, 0.103 | -0.081, 0.127 | -0.067, 0.071 | -0.017, 0.147 | -0.005,  -0.001 |
|  | P Value | 0.183 | 0.452 | 0.661 | 0.965 | 0.123 | 0.010 |
|  | Heterogeneity P | 0.287 |  |  |  | 0.417 |  |
|  | N Snps | 136 |  | F-Statistic | 97.6 |  |  |
| Eosinophil | Beta | -0.039 | 0.047 | 0.017 | -0.011 | -0.060 | 0.001 |
|  | 95% C.I. | -0.066,  -0.012 | -0.059, 0.153 | -0.054, 0.087 | -0.054, 0.032 | -0.117,  -0.003 | -0.001, 0.003 |
|  | P Value | 0.006 | 0.385 | 0.649 | 0.612 | 0.040 | 0.412 |
|  | Heterogeneity P | 0.014 |  |  |  | 0.013 |  |
|  | N Snps | 317 |  | F-Statistic | 121.3 |  |  |
| Lymphocytes | Beta | -0.011 | 0.048 | 0.042 | 0.024 | -0.013 | 5.52e-05 |
|  | 95% C.I. | -0.044, 0.022 | -0.074, 0.170 | -0.050, 0.134 | -0.023, 0.071 | -0.089, 0.063 | -0.002, 0.002 |
|  | P Value | 0.524 | 0.442 | 0.372 | 0.318 | 0.744 | 0.954 |
|  | Heterogeneity P | 2.67e-07 |  |  |  | 2.21e-07 |  |
|  | N Snps | 346 |  | F-Statistic | 96.1 |  |  |
| Monocytes | Beta | -0.013 | -0.002 | -0.015 | -0.014 | -0.013 | 2.29e-05 |
|  | 95% C.I. | -0.037, 0.011 | -0.073, 0.069 | -0.052, 0.022 | -0.051, 0.023 | -0.052, 0.026 | -0.002, 0.002 |
|  | P Value | 0.306 | 0.960 | 0.410 | 0.457 | 0.518 | 0.972 |
|  | Heterogeneity P | 9.38e-05 |  |  |  | 8.14e-05 |  |
|  | N Snps | 366 |  | F-Statistic | 160.9 |  |  |
| Neutrophils | Beta | -0.017 | -0.043 | -0.049 | -0.043 | 0.002 | -0.001 |
|  | 95% C.I. | -0.052, 0.018 | -0.174, 0.088 | -0.155, 0.057 | -0.096, 0.010 | -0.078, 0.082 | -0.002, 0.001 |
|  | P Value | 0.352 | 0.522 | 0.370 | 0.109 | 0.964 | 0.611 |
|  | Heterogeneity P | 0.016 |  |  |  | 0.015 |  |
|  | N Snps | 275 |  | F-Statistic | 86.3 |  |  |
| Platelets | Beta | 0.008 | 0.020 | 0.003 | 0.009 | 0.002 | 0.0002 |
|  | 95% C.I. | -0.014, 0.030 | -0.062, 0.102 | -0.044, 0.050 | -0.028, 0.046 | -0.041, 0.045 | -0.002, 0.002 |
|  | P Value | 0.474 | 0.633 | 0.903 | 0.638 | 0.911 | 0.750 |
|  | Heterogeneity P | 0.046 |  |  |  | 0.044 |  |
|  | N Snps | 418 |  | F-Statistic |  |  |  |

#IVW: Inverse Variance Weighted, C.I.: Confidence Interval MR-Egger Int: MR-Egger Intercept

**Supplementary Table 7: Two-Sample Mendelian Randomisation of Blood Counts (Exposure) and Pre-eclampsia and hypertensive disorders of pregnancy (Outcome).**

| **Exposure** | **Method** | **Analysis** | | | | | |
| --- | --- | --- | --- | --- | --- | --- | --- |
|  |  | **IVW** | **Simple Mode** | **Weighted Mode** | **Weighted Median** | **MR-Egger** | **MR-Egger Int** |
| Basophil | Odds Ratio | 0.987 | 0.845 | 0.845 | 0.913 | 0.931 | 1.002 |
|  | 95% C.I. | 0.871, 1.119 | 0.592, 1.204 | 0.675, 1.056 | 0.774, 1.076 | 0.729, 1.190 | 0.996, 1.008 |
|  | P Value | 0.841 | 0.352 | 0.141 | 0.275 | 0.571 | 0.589 |
|  | Heterogeneity P | 4.03e-06 |  |  |  | 3.45e-06 |  |
|  | N Snps | 153 |  | F-Statistic | 94.1 |  |  |
| Eosinophil | Odds Ratio | 0.973 | 1.006 | 1.021 | 0.985 | 0.905 | 0.998 |
|  | 95% C.I. | 0.903, 1.049 | 0.754, 1.341 | 0.861, 1.211 | 0.888, 1.093 | 0.905, 1.219 | 0.994, 1.002 |
|  | P Value | 0.468 | 0.966 | 0.806 | 0.779 | 0.520 | 0.245 |
|  | Heterogeneity P | 2.24e-08 |  |  |  | 2.54e-08 |  |
|  | N Snps | 365 |  | F-Statistic | 117.0 |  |  |
| Lymphocytes | Odds Ratio | 1.009 | 1.064 | 1.010 | 0.993 | 1.065 | 0.994 |
|  | 95% C.I. | 0.938, 1.085 | 0.785, 1.442 | 0.888, 1.359 | 0.892, 1.106 | 0.910, 1.246 | 0.994, 1.002 |
|  | P Value | 0.815 | 0.690 | 0.379 | 0.895 | 0.432 | 0.445 |
|  | Heterogeneity P | 0.0003 |  |  |  | 0.0003 |  |
|  | N Snps | 404 |  | F-Statistic | 98.3 |  |  |
| Monocytes | Odds Ratio | 0.995 | 0.944 | 0.979 | 0.969 | 1.049 | 0.998 |
|  | 95% C.I. | 0.935, 1.059 | 0.777, 1.146 | 0.895, 1.072 | 0.885, 1.060 | 0.946, 1.164 | 0.994, 1.002 |
|  | P Value | 0.888 | 0.556 | 0.641 | 0.487 | 0.368 | 0.220 |
|  | Heterogeneity P | 1.31e-10 |  |  |  | 1.56e-10 |  |
|  | N Snps | 417 |  | F-Statistic | 153.7 |  |  |
| Neutrophils | Odds Ratio | 0.894 | 0.925 | 0.911 | 0.908 | 0.766 | 1.004 |
|  | 95% C.I. | 0.823, 0.975 | 0.673, 1.271 | 0.730, 1.137 | 0.803, 1.028 | 0.632, 0.929 | 1.000, 1.008 |
|  | P Value | 0.012 | 0.633 | 0.411 | 0.125 | 0.007 | 0.080 |
|  | Heterogeneity P | 9.02e-07 |  |  |  | 1.48e-06 |  |
|  | N Snps | 317 |  | F-Statistic | 88.8 |  |  |
| Platelets | Odds Ratio | 0.974 | 1.010 | 1.010 | 1.029 | 1.053 | 0.997 |
|  | 95% C.I. | 0.913, 1.039 | 0.825, 1.236 | 0.902, 1.132 | 0.944, 1.122 | 0.933, 1.189 | 0.993, 1.001 |
|  | P Value | 0.432 | 0.926 | 0.868 | 0.511 | 0.403 | 0.142 |
|  | Heterogeneity P | 4.11e-15 |  |  |  | 6.21e-15 |  |
|  | N Snps | 472 |  | F-Statistic | 137.7 |  |  |

#IVW: Inverse Variance Weighted, C.I.: Confidence Interval, MR-Egger Int: MR-Egger Intercept

**References:**

[1. Warrington NM, Beaumont RN, Horikoshi M, Day FR, Helgeland Ø, Laurin C, et al. Maternal and fetal genetic effects on birth weight and their relevance to cardio-metabolic risk factors. Nat Genet. 2019;51:804–14.](http://paperpile.com/b/4KLcVF/rWNak)
